# TRIM28 Increases RFC4 Protein Expression to Promote Triple‐Negative Breast Cancer Progression

**DOI:** 10.1002/mco2.71043

**Published:** 2026-10-04

**Authors:** Cheng Guo, Li Liu, Lixiang Sun, Litong Liu, Han Wei, Yutong Yan, Fei Yu, Guangming Fu, Xianning Dong, Ceshi Chen, Chengqin Wang

**Affiliations:** ^1^ Department of Pathology The Affiliated Hospital of Qingdao University Qingdao Shandong China; ^2^ Department of Pathology School of Basic Medicine Qingdao University Qingdao Shandong China; ^3^ Department of Breast and Thyroid Surgery The First Affiliated Hospital of Chongqing Medical University Chongqing China; ^4^ The Third People's Hospital of Datong City Shanxi Province Shanxi China; ^5^ Yunnan Key Laboratory of Breast Cancer Precision Medicine Academy of Biomedical Engineering Kunming Medical University Kunming China; ^6^ Yunnan Key Laboratory of Breast Cancer Precision Medicine The Third Affiliated Hospital Kunming Medical University Kunming China

**Keywords:** ras‐associated protein 1, replication factor C subunit 4, tripartite motif‐containing 28, triple‐negative breast cancer, ubiquitination

## Abstract

Replication factor C subunit 4 (RFC4) dysregulation has been implicated in tumor progression and poor prognosis across multiple cancer types. Triple‐negative breast cancer (TNBC) is an aggressive subtype with limited treatment options and poor outcomes, underscoring the need for effective therapeutic targets. However, the role of RFC4 in TNBC progression and metastasis remains unclear. In the present study, integrated bioinformatics analyses, together with validation in 80 clinical TNBC samples, showed that RFC4 was markedly upregulated in TNBC. In vitro functional assays and experiments in nude mouse models further demonstrated that elevated RFC4 expression contributed to TNBC progression (*p* < 0.05). Mechanistically, the upstream transcription factor TRIM28 (Tripartite motif‐containing 28) bound to the BS2 and BS3 sites within the *RFC4* promoter region, thereby activating *RFC4* transcription and increasing its expression. Furthermore, TRIM28 interacted with RFC4 and assembled K29‐linked ubiquitin chains at K205 and K217, enhancing RFC4 ubiquitination and protein stability in a manner dependent on TRIM28 ubiquitin ligase activity (*p* < 0.05). Finally, we showed that RFC4 activates the Ras‐associated protein 1 (Rap1)‐PI3K–AKT pathway in a ubiquitination‐dependent manner, thereby enhancing TNBC cell proliferation and metastasis. Together, these findings suggest that the TRIM28–RFC4 axis may represent a promising therapeutic target for TNBC.

## Graphical abstract

RFC4 dysregulation has been implicated in tumor progression and poor prognosis across multiple cancer types. Triple‐negative breast cancer (TNBC) is an aggressive breast cancer subtype with limited therapeutic options and unfavorable clinical outcomes, highlighting the need to identify effective therapeutic targets. However, the role of RFC4 in TNBC progression and metastasis remains unclear. In the present study, integrated bioinformatics analyses and validation in 80 TNBC clinical samples showed that RFC4 was markedly upregulated in TNBC. Functional assays in vitro and in nude mouse models further demonstrated that elevated RFC4 expression contributed to TNBC progression (*p* < 0.05). Mechanistically, the upstream transcription factor TRIM28 bound to the BS2 and BS3 sites within the RFC4 promoter region, thereby activating RFC4 transcription and upregulating its expression. Furthermore, TRIM28 interacted with RFC4 and assembled K29‐linked ubiquitin chains at residues K205 and K217, enhancing RFC4 ubiquitination and protein stability in a manner dependent on its ubiquitin ligase activity (*p* < 0.05). Finally, we showed that RFC4 activates the Rap1–PI3K–AKT pathway in a ubiquitination‐dependent manner, thereby enhancing TNBC cell proliferation and metastasis. Together, these findings suggest that the TRIM28–RFC4 axis may represent a promising therapeutic target for TNBC.

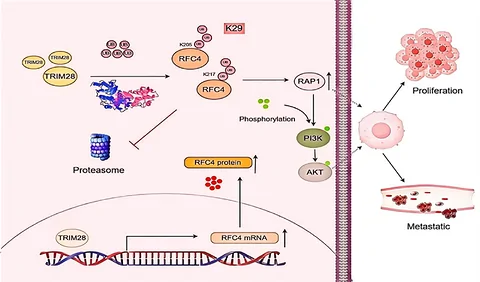

## 1 Introduction

Breast cancer is a leading contributor to cancer‐related mortality among women worldwide [1]. Among breast cancer subtypes, ER−, PR−, and HER2− (triple‐negative) breast cancer (TNBC) accounts for approximately 15%–20% of cases [2] and represents the most clinically aggressive subtype with the poorest outcomes owing to the lack of targetable hormone receptors and limited effective therapeutic options [2, 3, 4]. Despite significant advances in TNBC treatment, tumor heterogeneity and biological complexity remain major therapeutic challenges. Therefore, identifying effective targets to inhibit TNBC growth and metastasis remains an urgent need [5].

Replication factor C (RFC) is a structure‐specific DNA‐binding complex involved in DNA replication and DNA polymerase coordination [6, 7] and plays a crucial role in various malignancies [8, 9, 10]. The complex comprises five conserved subunits: the large subunit RFC1 and the small subunits RFC2–RFC5 [6]. Among these, RFC4 is the core component required for assembly of the RFC complex [11]. Multiple bioinformatics analyses have shown that RFC4 is upregulated in various tumor types and that elevated RFC4 expression is associated with poor clinical outcomes [12, 13]. Mechanistic studies have shown that RFC4 regulates DNA replication through its interaction with proliferating cell nuclear antigen (PCNA), thereby affecting tumor stemness and radio resistance. Furthermore, RFC4 can promote ER transactivation by forming an RFC4‐LPP chimeric transcript, thereby promoting mouse breast cancer progression [14]. To date, research on RFC4 in breast cancer, particularly TNBC, remains limited.

The tripartite motif (TRIM) family is defined by a conserved RING finger domain, one or more B‐box domains, and a coiled‐coil region [15] and represents the largest class of single‐protein RING‐type E3 ubiquitin ligases [16]. TRIM28, also known as TIF1β/KAP1, regulates gene transcription and DNA damage responses [17]. It contains an N‐terminal RING domain, two B‐box domains, a coiled‐coil region, a PxVxL motif, and C‐terminal PHD and bromodomains, supporting its role as a classical RING‐type E3 ubiquitin ligase [18]. TRIM28 also forms a complex with KRAB‐domain‐containing ZNF689, which directly binds the LINE‐1 promoter [19]. This interaction induces H3K9me3‐mediated transcriptional silencing, which exacerbates genomic instability and promotes intratumoral heterogeneity [19]. In addition, TRIM28 can regulate PD‐L1 ubiquitination and SUMOylation, thereby stabilizing PD‐L1 and modulating the tumor immune microenvironment [20].

In this study, we demonstrated that RFC4 is upregulated in TNBC and is associated with poor prognosis, supporting its potential as a diagnostic marker. Mechanistically, TRIM28 enhances *RFC4* transcription through promoter binding and assembles K29‐linked polyubiquitin chains on the RFC4 to stabilize it. We further found that RFC4 activates the RAP1‐PI3K‐AKT axis. Together, our findings identify the TRIM28–RFC4–RAP1 axis as a key regulator of TNBC progression and metastasis, providing promising targets for the treatment of TNBC.

## 2 Result

### 2.1 RFC4 Is Upregulated in TNBC Tissues and Predicts Poor Overall Survival

We first examined RFC4 expression in breast cancer using public datasets. In the TCGA database, RFC4 expression was significantly higher in breast cancer tissues than in normal tissues (Figure 1A,B). This finding was further supported by analyses of the GSE38959 and CPTAC datasets (Figure 1C; Figure S1A). Comparative analyses of the TCGA, CPTAC, and METABRIC datasets further showed that RFC4 expression was markedly elevated in TNBC compared with other breast cancer subtypes (Figure 1D–F).

**FIGURE 1:**
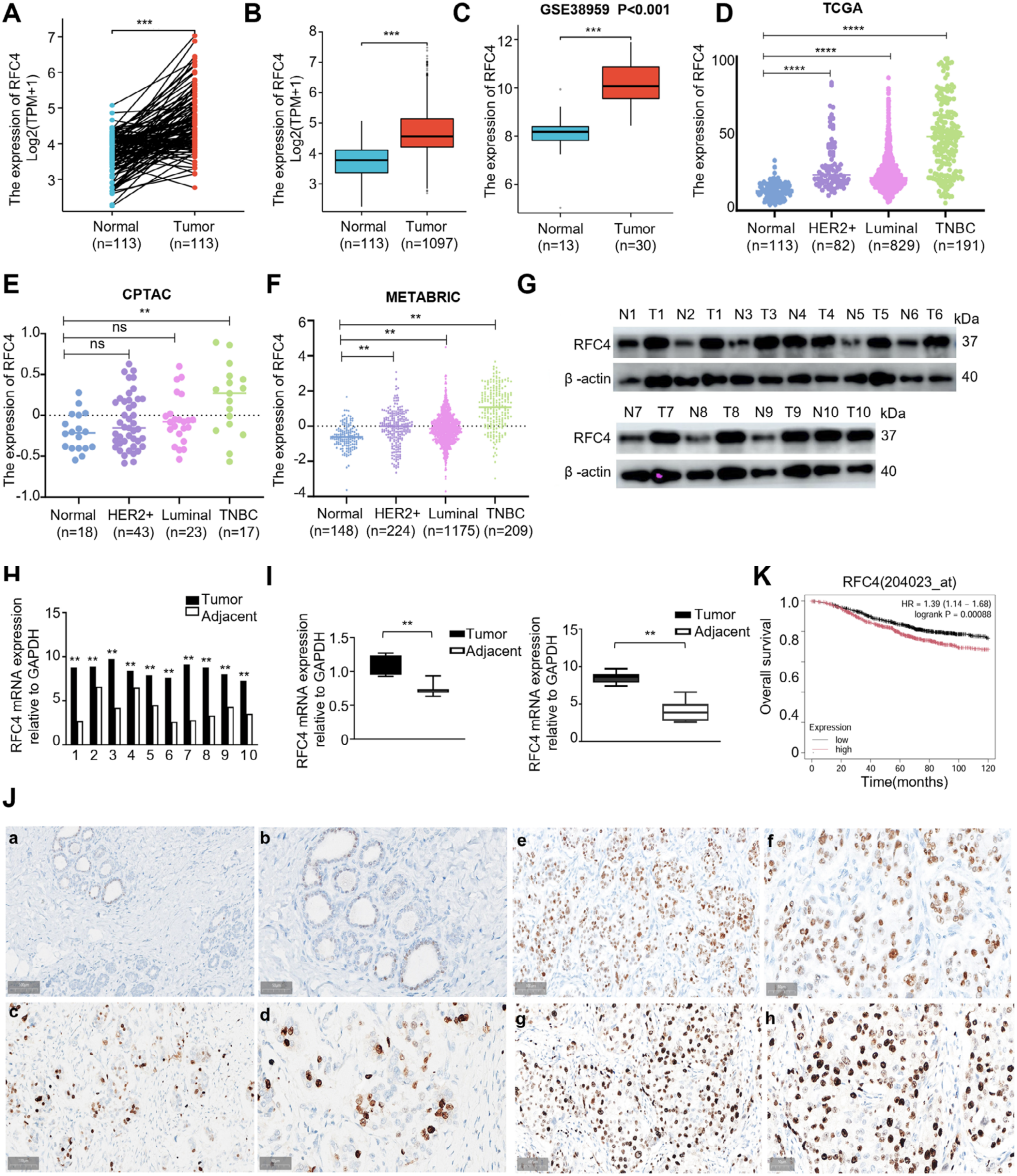
RFC4 is upregulated in TNBC tissues and predicts poor overall survival. (A) RFC4 levels in paired TNBC tissues and normal tissues in TCGA. (B) RFC4 levels in unpaired TNBC tissues and normal tissues in TCGA. (C) RFC4 levels in TNBC tissues and normal tissues in GSE38959. (D–F) TCGA, CPTAC, and METABRIC databases showed that the expression level of RFC4 is higher in TNBC compared to other subtypes. (G) RFC4 protein expression levels in TNBC were higher than that in normal tissues. T: tumor, N: normal tissue. (H and I) The mRNA expressions of RFC4 were measured by qRT‐PCR. **p < 0.01. (J) Representative IHC images of RFC4 expression. (a, b) IHC findings showed that the expression level of RFC4 in TNBC tissues was higher than that in adjacent tissues. (c, d) RFC4 was located in the cell nucleus. (e, f) The immuno‐staining is strongly positive in lymph node metastasis than in primary tumor tissues. (g, h) The RFC4 expression level was higher in the primary tumors with lymph node metastasis than that without lymph node metastasis; Scale bar, 50 and 100 µm. (K) Kaplan–Meier analysis of the OS of TNBC patients with high and low RFC4 expression.

To validate these findings, we collected TNBC tissues and matched adjacent normal tissues. RFC4 expression was examined in 10 paired TNBC and adjacent normal tissues by western blotting and RT‐qPCR. The results confirmed that RFC4 was markedly upregulated in TNBC tissues (Figure 1G–I). RFC4 protein and mRNA levels were also assessed in normal breast epithelial cells, TNBC cell lines, and non‐TNBC breast cancer cell lines. Consistently, RFC4 expression was significantly elevated in TNBC cells (Figure S1B–D). In addition, we conducted immunohistochemical (IHC) analysis of 80 samples of TNBC and assessed the association between RFC4 expression and patient clinicopathological features. Elevated RFC4 expression was significantly associated with both tumor recurrence and lymph node metastasis (Table 1). IHC staining indicated predominant nuclear localization of RFC4 (Figure (1J a and b)). Compared with adjacent normal tissues, TNBC tissues exhibited significantly higher RFC4 expression (Figure (1J c and d)). In addition, RFC4 staining was stronger in primary tumors with lymph node metastasis than in those without lymph node metastasis (Figure (1J e–h)). Kaplan–Meier plotter analysis showed that high RFC4 expression was associated with poor overall survival in patients with TNBC (Figure 1K).

**TABLE 1:** Association between RFC4 in TNBC and patient characteristics.

Variables	*n*	Mean ± SD	*Z* value	*p* value
Age				
≤ 60 years	52	5.789 ± 0.776	−1.767	0.077
> 60 years	28	5.714 ± 0.763		
Tumor size (cm)				
≤ 2	46	5.696 ± 0.813	−1.000	0.317
> 2	34	5.853 ± 0.702		
Tumor grade				
II	27	5.407 ± 0.747	−1.689	0.091
III	53	5.943 ± 0.718		
Adjacent tissues	80	4.563 ± 0.653	−7.126	<0.001*
Carcinoma	80	5.763 ± 0.767		
Lymph node metastasis				
Negative	55	5.473 ± 0.663	−2.887	0.004*
Positive	25	6.400 ± 0.577		
Vascular invasion				
Absence	65	5.769 ± 0.825	−0.970	0.332
Presence	15	5.733 ± 0.458		
Tumor recurrence				
Negative	57	5.509 ± 0.658	−2.496	0.013*
Positive	23	6.391 ± 0.656		

*Significant at < 0.05.

### 2.2 RFC4 Promotes TNBC Cell Growth and Metastasis

Next, we explored the function of RFC4 in TNBC cell lines. As shown in Figure S2A–C, RFC4 expression was high in MDA‐MB‐231 and MDA‐MB‐468 cell lines. Accordingly, we stably knocked down RFC4 in these two cell lines. In parallel, we overexpressed RFC4 in BT‐549 and Hs578T cell lines (Figure 2A–C; Figure S2D–G). CCK‐8 and colony formation assays demonstrated that RFC4 upregulation increased TNBC cell proliferation, whereas RFC4 depletion produced the opposite effect (Figure 2D–G; Figure S2H–M). Consistently, RFC4 knockdown in MDA‐MB‐231 cells suppressed xenograft tumor growth in vivo (Figure 2H,I). We then evaluated the role of RFC4 in TNBC cell migration and metastasis. Wound‐healing assays showed that RFC4 overexpression promoted cell migration, whereas RFC4 silencing significantly inhibited tumor cells migration (Figure 2J–K). Transwell assay yielded similar results (Figure 2M–N; Figure S2N–S). We injected MDA‐MB‐231 and MDA‐MB‐231‐shRFC4 cells via the tail vein. RFC4 knockdown significantly reduced the number of lung metastases (Figure 2N–Q).

**FIGURE 2:**
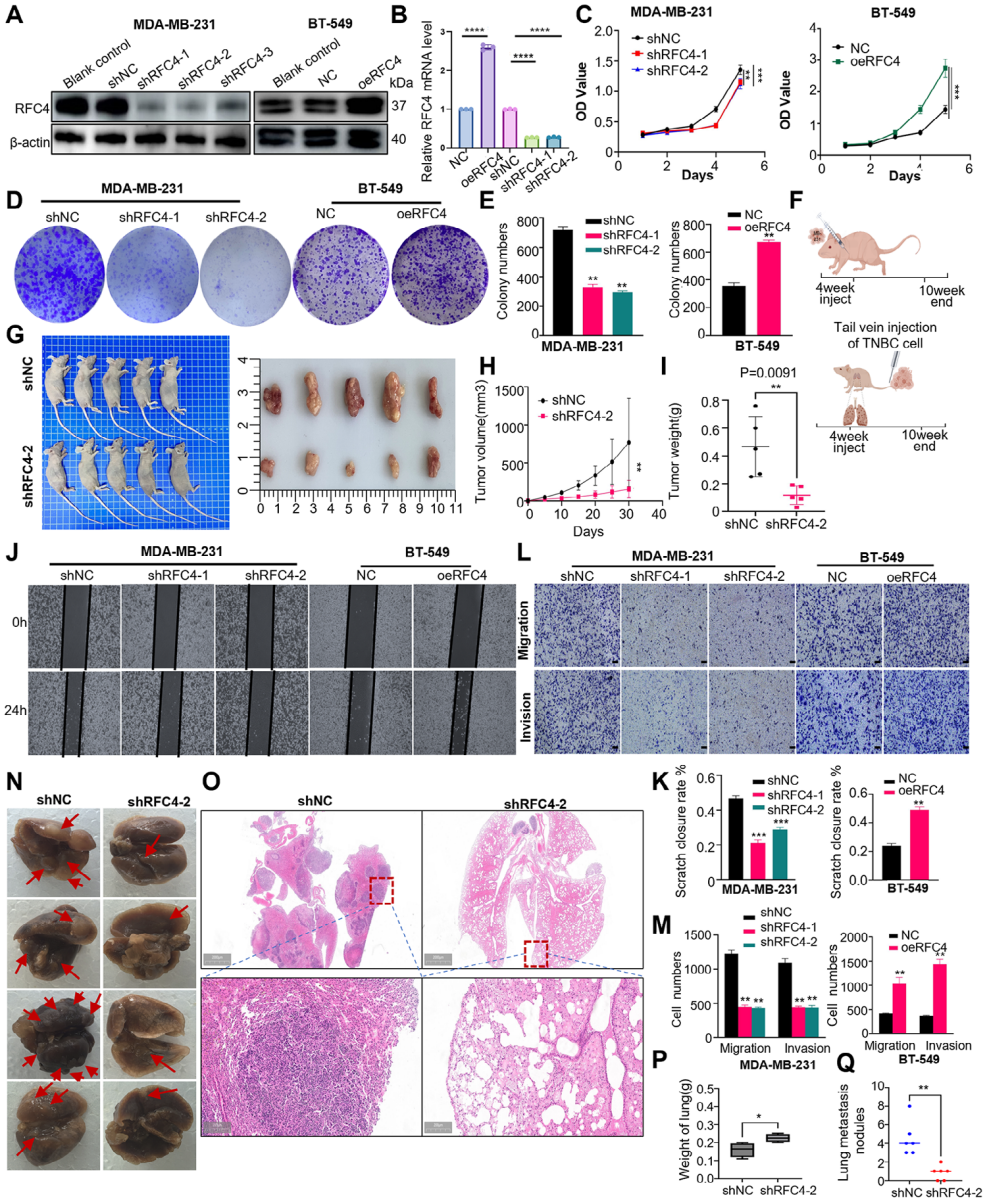
RFC4 promotes TNBC cell growth and metastasis. (A and B) Verification of transfection efficiency for RFC4 knockdown and overexpression lentiviruses at both the protein and mRNA levels. (C) Cell viability was assessed using the CCK‐8 assay. (D and E) Colony formation assays in MDA‐MB‐231 and BT549 cells stably expressing shRFC4 or oeRFC4. (F) Schematic representation of the nude mouse xenograft experiment. (G) Representative images of tumors dissected from mice injected with RFC4 knockdown or control MDA‐MB‐231 cells. (H and I) Growth curves and final weights of xenograft tumors. (J and K) Wound healing assays to evaluate cell migration. (L and M) Transwell assays to assess migration and invasion capabilities; Scale bar, 50 µm. (N and O) Lung metastasis model used to examine in vivo metastatic potential under different experimental conditions. Scale bar, 200 and 2000 µm. (P and Q) Quantification of lung weight (P) and number of metastatic nodules (Q). **p < 0.01, ***p < 0.001.

### 2.3 TRIM28 Activates *RFC4* Gene Transcription

To explore the mechanism underlying RFC4 overexpression in TNBC, we used UCSC, Human TF, GRAD, and PROMO to predict the upstream transcription factors of *RFC4* (Figure 3A). Four potential upstream transcription factors, including TBP, USF2, POU2F1, and TRIM28, were identified. TIMER2.0 analysis showed that TRIM28 mRNA expression was positively correlated with RFC4 expression in TNBC (Figure 3B; Figure S3A–C). TCGA and CPTAC datasets further indicated that TRIM28 was upregulated in breast cancer tissues, particularly in TNBC (Figure S3D–E). Consistently, western blotting and RT‐qPCR confirmed significantly higher TRIM28 protein and mRNA levels in TNBC cell lines than in MCF‐10A cells. (Figure 3C,D).

**FIGURE 3:**
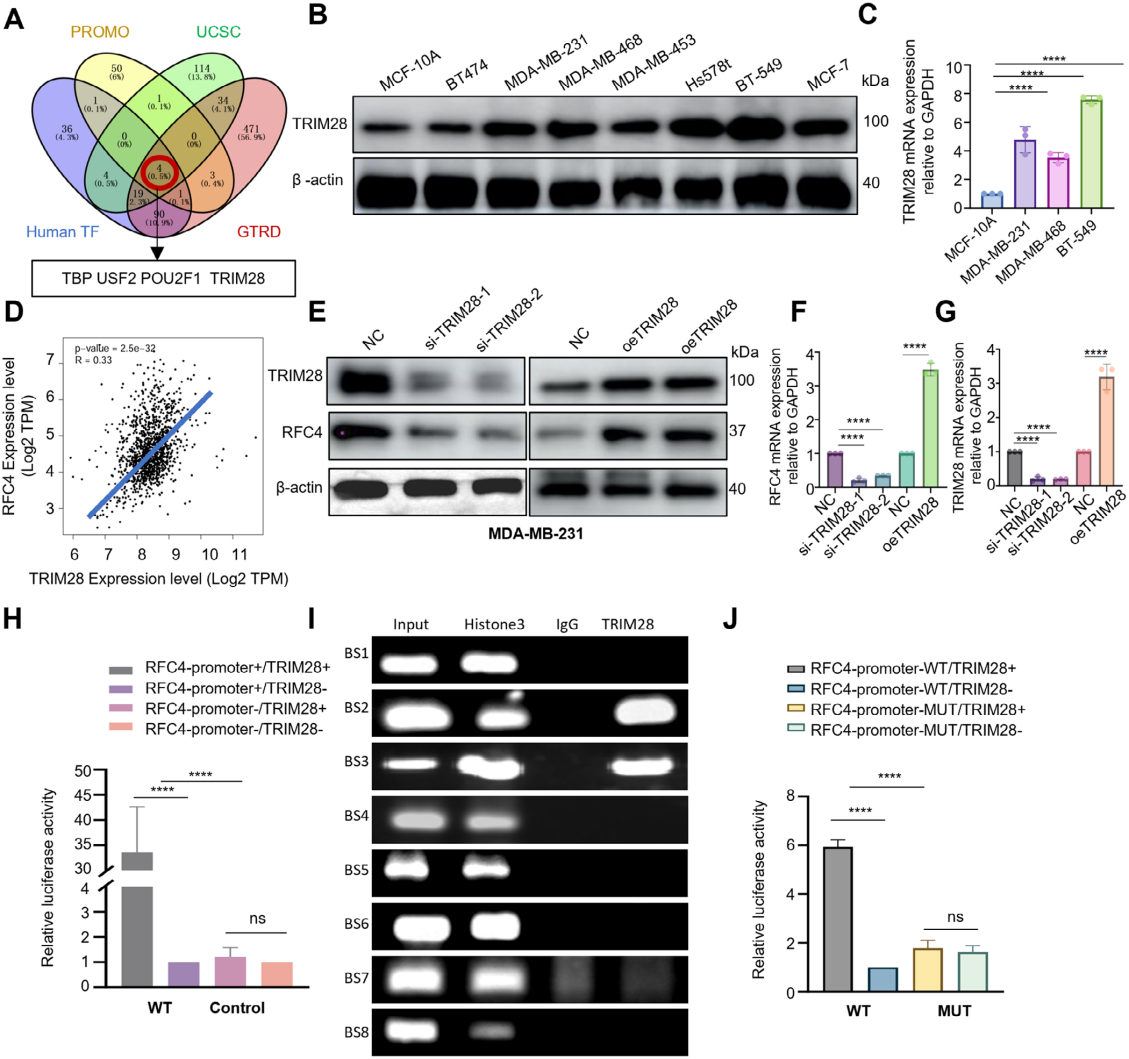
TRIM28 activates *RFC4* gene transcription. (A) Venn diagram of proteins predicted to bind the *RFC4* promoter region using the UCSC, PROMO, GTRD, and human TF databases. (B) Correlation analysis between RFC4 and TRIM28 using TIMER 2.0. (C and D) Immunoblotting analysis of TRIM28 expression in TNBC cells. (E) Expression levels of TRIM28 and RFC4 in TNBC cells. (F and G) qRT‐PCR analysis of *TRIM28* and *RFC4* mRNA expression. (H) Dual‐luciferase reporter assay assessing the effect of TRIM28 on *RFC4* promoter activity. (I) ChIP assay identifying the binding sites of TRIM28 within the *RFC4* promoter region. (J) Dual‐luciferase reporter assay showing that the BS2 and BS3 sites displayed transcriptional activity.

Subsequently, we tested whether TRIM28 promotes RFC4 expression in TNBC cells. TRIM28 depletion reduced RFC4 protein and mRNA levels, whereas TRIM28 overexpression markedly increased both (Figure 3E–G). In contrast, RFC4 overexpression or knockdown did not significantly alter TRIM28 expression (Figure S3F–G). Based on these findings, we hypothesized that TRIM28 promotes *RFC4* transcription. To test this hypothesis, we constructed *RFC4* gene promoter‐reporter plasmids and performed dual‐luciferase assays. As expected, TRIM28 overexpression significantly increased luciferase activity, indicating that TRIM28 enhances the *RFC4* gene promoter activity (Figure 3H). Human TFBS prediction identified eight potential TRIM28 binding sites within the *RFC4* promoter region (Figure S3H,I). Chromatin immunoprecipitation (ChIP) experiments demonstrated that TRIM28 binds to the BS2 and BS3 sites within the RFC4 promoter (Figure 3I). When the BS2 and BS3 sites were mutated, TRIM28 failed to activate the RFC4 gene promoter (Figure 3J). In summary, our findings suggest that TRIM28 activates *RFC4* gene promoter to enhance its transcription.

### 2.4 TRIM28 Specifically Interacts With RFC4

STRING database analysis predicted an interaction between TRIM28 and RFC4 (Figure S4A). We co‐transfected Flag‐RFC4 and Myc‐TRIM28 into HEK293T cells to examine the interaction between the two proteins. As expected, exogenous TRIM28 and RFC4 proteins interacted with each other (Figure S4B). We further validated the interaction between endogenous TRIM28 and RFC4 proteins in HEK293T and MDA‐MB‐231 cells through co‐immunoprecipitation (CO‐IP) (Figure 4A). Immunofluorescence staining further showed that TRIM28 and RFC4 were co‐localized in the nucleus (Figure 4B). Additionally, subcellular fractionation showed that TRIM28 and RFC4 were predominantly localized in the nucleus (Figure 4C).

**FIGURE 4:**
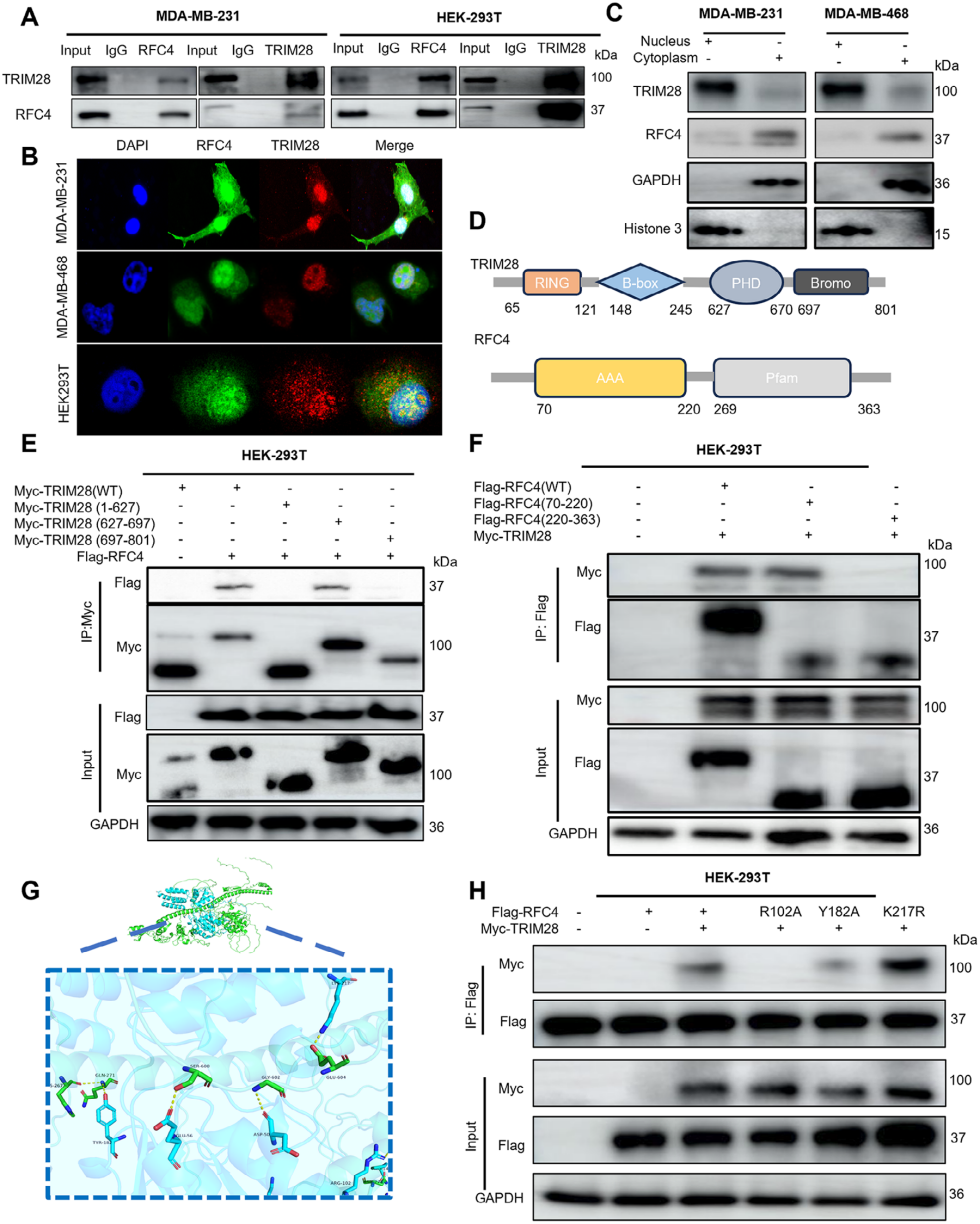
TRIM28 specifically interacts with RFC4. (A) Co‐IP analysis confirming the interaction between TRIM28 and RFC4 in TNBC and HEK293T cells. (B) Immunofluorescence images showing the colocalization of TRIM28 and RFC4 in MDA‐MB‐231, MDA‐MB‐468, and HEK293T cells stably co‐expressing RFC4 (green) and TRIM28 (red). Nuclei were counterstained with DAPI (blue). (C) Western blot analysis of TRIM28 and RFC4 expression in the cytoplasmic and nuclear fractions of TNBC cells. (D) Schematic representation of full‐length TRIM28 and RFC4 and their truncated mutants. (E) Co‐IP analysis examining the interaction between Flag‐RFC4 and full‐length or truncated Myc‐TRIM28 mutants in TNBC cells. (F) Co‐IP analysis examining the interaction between Myc‐TRIM28 and full‐length or truncated Flag‐RFC4 mutants in TNBC cells. (G) Predicted binding mode of RFC4 with the AlphaFold‐generated homology model of TRIM28. RFC4 is shown as blue sticks; hydrogen bonds are indicated by dashed lines, and key amino acid residues involved in binding are shown as yellow sticks. (H) Western blot analysis of wild‐type and mutant Flag‐RFC4 proteins following Myc‐TRIM28 pull‐down in HEK293T cells.

To determine which domains of TRIM28 interact with RFC4, we constructed truncated TRIM28 proteins (Figure 4D). As shown in Figure 4E, the PHD domain (627–697 aa) of TRIM28 strongly bound to RFC4. The AAA domain (70–220 aa) of RFC4 was the key structural domain for the interaction (Figure 4F). To further investigate the key binding sites between TRIM28 and RFC4, we employed molecular docking. Three‐dimensional structural analysis revealed that TRIM28 (green) and RFC4 (blue) could stably associate with each other (Figure 4G). Based on the prediction results (Figure S4C), we mutated RFC4 at R102, Y182, and K217, and found that the R102A and Y182A mutants failed to interact with TRIM28 (Figure 4H).

### 2.5 TRIM28 Stabilizes RFC4 in TNBC Cells

Given the established role of TRIM28 as an E3 ubiquitin ligase in regulating protein stability [18], we investigated whether TRIM28 regulates RFC4 stability. TRIM28 overexpression markedly extended the half‐life of RFC4 (Figure 5A). Treatment with MG132, but not the lysosomal inhibitor chloroquine (CQ), increased RFC4 protein levels in a time‐dependent manner, indicating that RFC4 is primarily degraded through the proteasomal pathway (Figure S4D–F).

**FIGURE 5:**
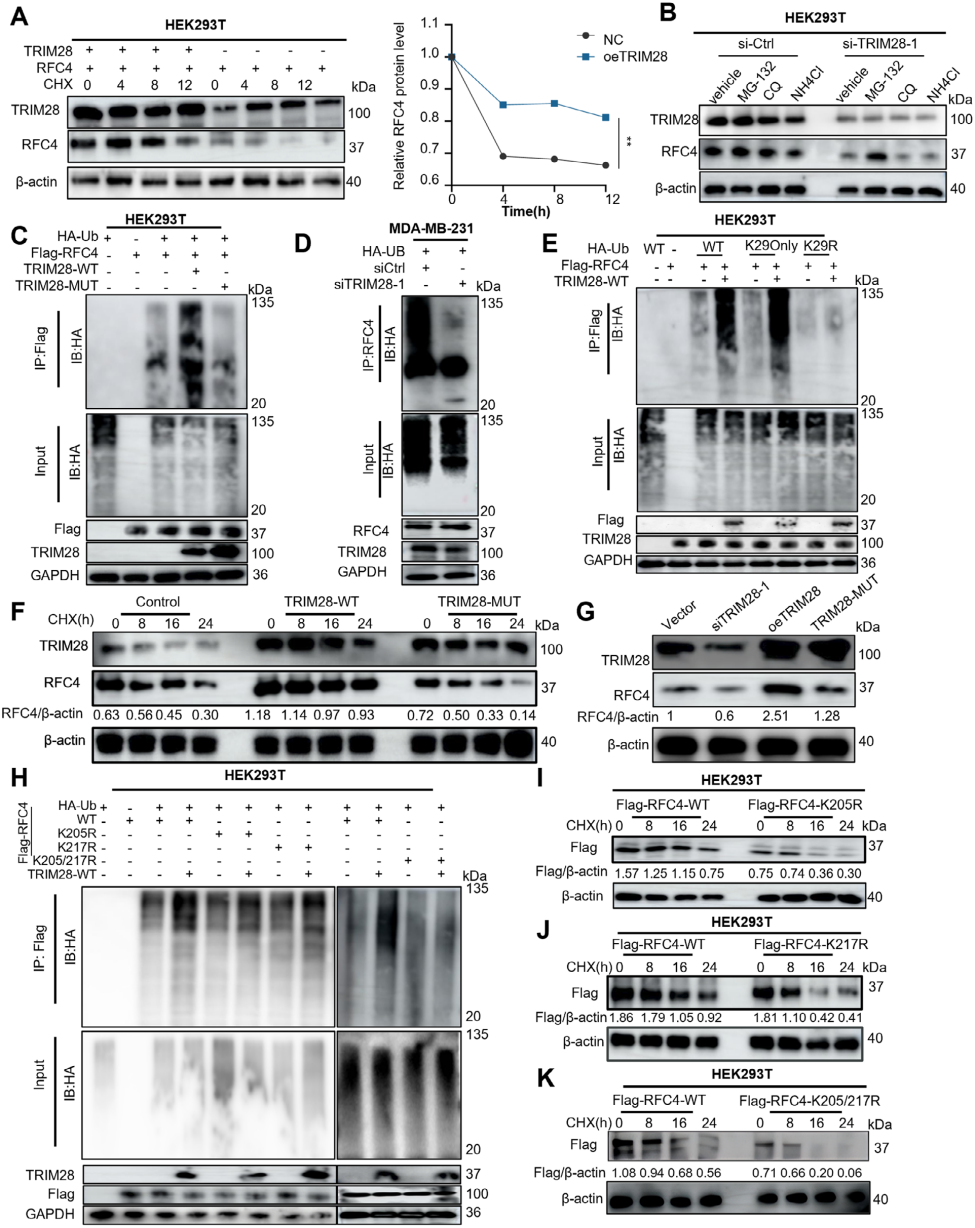
TRIM28 ubiquitinates RFC4 with K29‐linked polyubiquitin chains. (A) MDA‐MB‐231 cells were transduced with lentiviruses carrying empty vector or oeTRIM28 and treated with cycloheximide (CHX) for 0, 4, 8, and 12 h. (B) TRIM28 knockdown promoted RFC4 protein degradation through the proteasome pathway. (C) Analysis of the ubiquitin chain types on RFC4 affected by TRIM28. Ubiquitinated proteins were pulled down using anti‐Flag agarose under denaturing conditions from HEK293T cells transfected with the indicated constructs and analyzed by western blotting with the indicated antibodies. At 24 h after transfection, cells were treated with MG132 (10 µM) for an additional 4 h. (D) Analysis of endogenous RFC4 ubiquitination in MDA‐MB‐231 cells following siRNA‐mediated TRIM28 knockdown. (E) TRIM28‐mediated K29‐linked polyubiquitination of RFC4. (F) Effect of mutation of the TRIM28 enzymatic active site on the half‐life of RFC4. (G) Quantitative analysis of RFC4 protein levels after mutation of the TRIM28 enzymatic active site. (H) TRIM28 ubiquitinated RFC4 at K205 and K217. (I–K) Analysis of the half‐lives of Flag‐RFC4 wild‐type, K205R, K217R, and K205/217R mutant proteins in HEK293T cells.

Furthermore, we treated TRIM28 knockdown cells with MG132 and found that MG132 partially rescued the reduction in RFC4 caused by TRIM28 knockdown (Figure 5B). In contrast, the lysosome inhibitors CQ and NH_4_Cl did not rescue this effect (Figure 5B).

### 2.6 TRIM28 Ubiquitinates RFC4 With K29‐Linked Polyubiquitin Chains

Next, we examined whether TRIM28 ubiquitinates RFC4. As expected, TRIM28 increased RFC4 polyubiquitination in HEK293T cells in an E3 ubiquitin ligase activity‐dependent manner (Figure 5C). Consistently, TRIM28 knockdown significantly reduced polyubiquitination of endogenous RFC4 in MDA‐MB‐231 cells (Figure 5D). Moreover, TRIM28‐mediated RFC4 ubiquitination occurred in both the nucleus and cytoplasm (Figure S5A). Since TRIM28 stabilizes RFC4, we further explore the ubiquitin linkage type mediated by TRIM28 using seven ubiquitin mutants, including K6R, K11R, K27R, K29R, K33R, K48R, and K63R. As a result, The K29R mutant significantly reduced TRIM28‐induced RFC4 ubiquitination, while other mutants did not significant effect (Figure S5B). We further validated this result by using the K29 only ubiquitin plasmid, in which K29 is the only lysine retained. Under these conditions, TRIM28 primarily assembled K29‐linked polyubiquitin chains on RFC4 (Figure 5E). Interestingly, TRIM28 increased both RFC4 expression and protein stability in an E3 ubiquitin ligase activity‐dependent manner (Figure 5F–G).

To further identify the RFC4 ubiquitination sites targeted by TRIM28, we first investigated which RFC4 domain was primarily ubiquitinated and found that TRIM28 ubiquitinated the AAA domain (Figure S6A). We then mutated all 12 Iysine (K) residues in the RFC4 AAA domain to arginine (R) and evaluated their ubiquitination levels (Figure S6B). These experiments identified K205 and K217 as potential TRIM28‐mediated ubiquitination sites on RFC4 (Figure S6C). Mutation of the K205 and K217 residues indeed abolished the TRIM28‐induced RFC4 ubiquitination (Figure 5H). To test whether TRIM28‐mediated RFC4 ubiquitination increased protein stability, we compared the protein stability of K205R, K217R, and wild‐type (WT) in HEK293T cells. Compared to WT, RFC4‐K205R, ‐K217R, and both‐K205R/K217R mutants markedly decreased the protein stability (Figure 5I–K). Thus, TRIM28 promotes the stabilization of RFC4 protein by catalyzing K29‐linked ubiquitination at K205 and K217.

### 2.7 TRIM28 Promoted TNBC Proliferation and Metastasis by Upregulating RFC4 in Part

To assess whether RFC4 contributes to TRIM28‐mediated TNBC progression, we overexpressed TRIM28 while concurrently knocking down RFC4 in MDA‐MB‐231 and MDA‐MB‐468 cells (Figure 6A,B). As expected, TRIM28 promoted TNBC cell proliferation, migration, and invasion, whereas RFC4 knockdown partially reverses these TRIM28‐induced phenotypes (Figure 6C–I).

**FIGURE 6:**
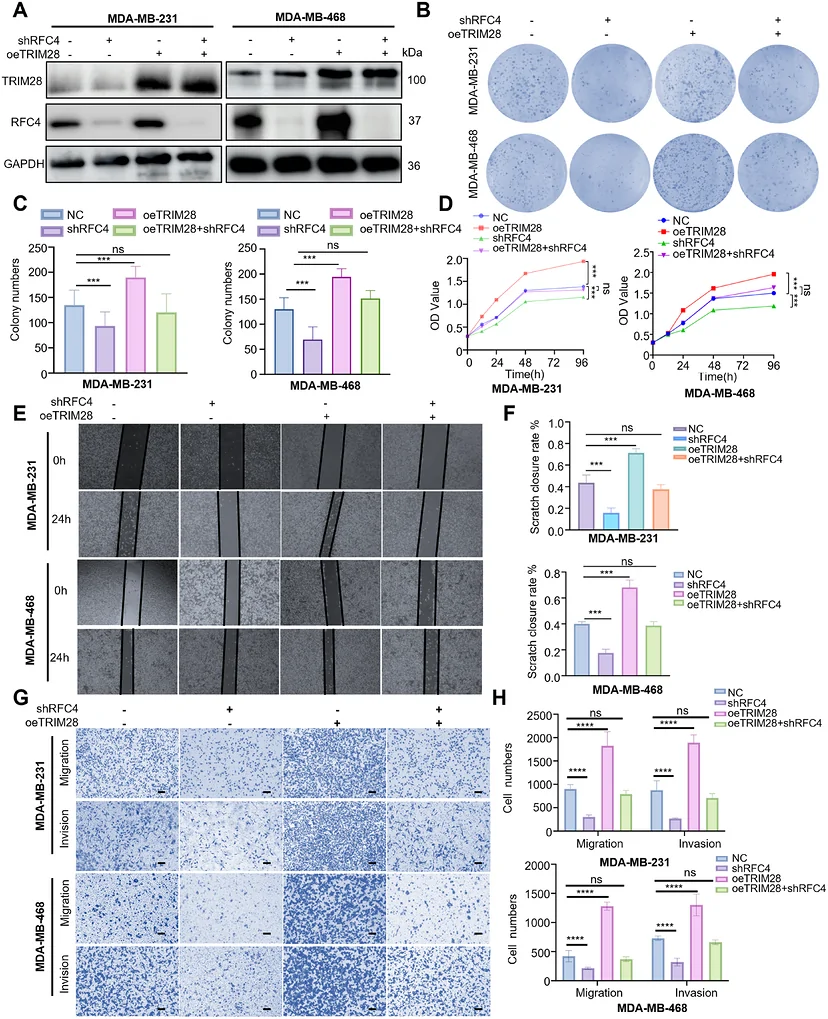
**T**RIM28 promoted TNBC proliferation and metastasis by upregulating RFC4 in part. (A) Protein and mRNA levels of TRIM28 and RFC4 were verified in MDA‐MB‐231 and MDA‐MB‐468 cells after lentiviral transduction. (B and C) Colony formation assays were performed to determine the effects of TRIM28 knockdown and overexpression on cell proliferation. (D) Cell viability was assessed using the CCK‐8 assay. (E and F) Wound healing assays were used to evaluate cell migration ability. (G and H) Transwell assays were performed to assess cell migration and invasion abilities; Scale bar, 50 µm.

### 2.8 RFC4 Promotes TNBC via the RAP1‐PI3K‐AKT Signal Pathway

RNA‐seq was performed in RFC4‐overexpressing TNBC cells to investigate downstream mechanisms of RFC4. A total of 412 genes were upregulated and 359 genes were downregulated after RFC4 overexpression (Figure S7A). KEGG and GSEA analyses indicated that RFC4 was significantly associated with the RAP1 pathway (Figure S7B,C). RAP1 is a small GTPase [21]. By analyzing genes enriched in the RAP1 pathway (Figure S7D) and the RAP1 signaling network (Figure S7E), we further examined three candidate pathways, including PI3K–AKT, MAPK, and Wnt/β‐catenin. RFC4 overexpression exerted the strongest regulatory effect on the PI3K–AKT pathway compared with the other pathways (Figure S7F).

Subsequently, we examined the activation of the RAP1‐PI3K‐AKT pathway. As shown in Figure 7A, RFC4 overexpression activated the RAP1 pathway, as indicated by the upregulation of RAP1, p‐PI3K, and p‐AKT. Consistently, RFC4 knockdown significantly inhibited this pathway (Figure S7G). Similarly, IHC analysis of xenograft tumors showed reduced expression of RFC4, RAP1, p‐PI3K, and p‐AKT after RFC4 silencing compared with the control group (Figure S8A).

**FIGURE 7:**
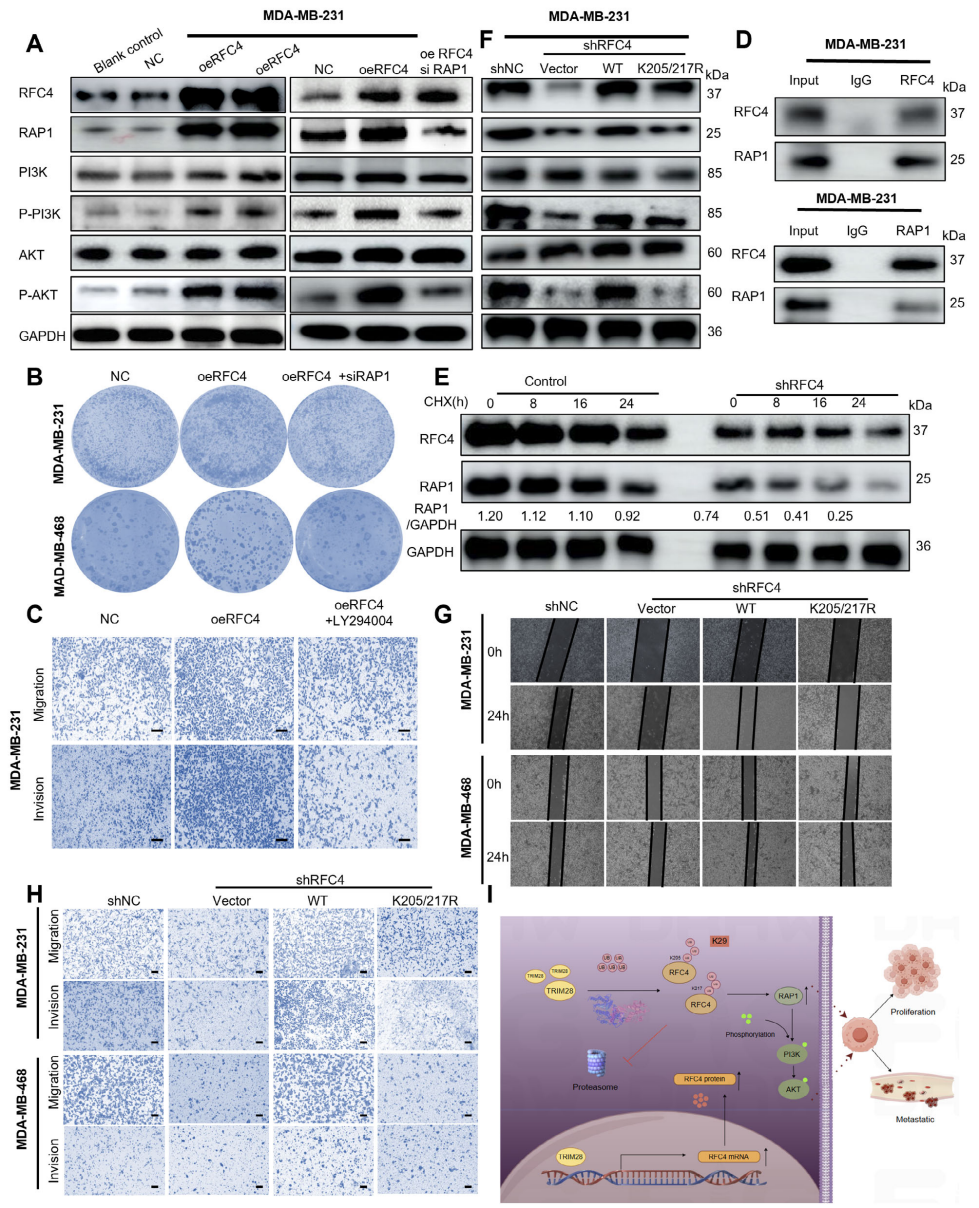
RFC4 promotes TNBC via the RAP1‐PI3K‐AKT signal pathway. (A) Immunoblot analysis of RAP1‐PI3K‐AKT pathway‐related proteins in MDA‐MB‐231 cells stably overexpressing RFC4, with the effect of RFC4 on the PI3K‐AKT pathway further verified by RAP1 knockdown using siRNA. (B) Colony formation assay to assess the effects of RFC4 and RAP1 on cell proliferation. (C) Transwell assays to evaluate cell migration and invasion abilities. Scale bar: 50 µm. (D) Co‐IP analysis confirming the interaction between RFC4 and RAP1 in TNBC cells. (E) Analysis of RAP1 protein half‐life following RFC4 knockdown. (F) Effects of RFC4 ubiquitination status on the RAP1 signaling pathway. (G and H) Effects of ubiquitin site mutations in RFC4 on cell migration and invasion. Scale bar, 50 µm. (I) Schematic diagram illustrating the mechanism by which TRIM28 transcriptionally activates RFC4, promotes its ubiquitination‐mediated stability, and subsequently activates the RAP1‐PI3K‐AKT pathway.

We further investigated whether RFC4 functions through RAP1. Activation of the PI3K‐AKT pathway by RFC4 overexpression was abolished by RAP1 knockdown (Figure 7A). Consistent with this finding, the pro‐proliferative and pro‐migratory effects of RFC4 were reversed by RAP1 knockdown (Figure 7B; Figure S8B,C). We next assessed whether RFC4 promotes TNBC cell proliferation and migration through the PI3K‐AKT pathway. The PI3K inhibitor LY294004 significantly suppressed RFC4‐induced proliferation, migration, and invasion induced in MDA‐MB‐231 cells (Figure 7C; Figure S8D,E).

To characterize the mechanism by which RFC4 upregulates RAP1 expression, we measured *RAP1* mRNA levels and found that RFC4 did not affect *RAP1* mRNA levels (Figure S8F). We further demonstrated that RFC4 interacted with RAP1 and increased RAP1 protein stability (Figure 7D,E; Figure S8G,H). Interestingly, numerous studies have reported that TRIM28 can promote cancer by activating the PI3K‐AKT pathway [22]. We further examined whether TRIM28 regulates the RAP1–PI3K–AKT pathway through RFC4. TRIM28 overexpression activated this pathway in TNBC cells, whereas LY294002 treatment suppressed TRIM28‐induced cell proliferation and migration (Figure S8I), suggesting that PI3K–AKT signaling contributes to the oncogenic effects of TRIM28 (Figure S8J–M).

We next assessed whether RFC4 ubiquitination was required for RAP1–PI3K–AKT pathway activation. Unlike wild‐type RFC4, the ubiquitination‐deficient K205R/K217R mutant failed to elevate RAP1, p‐PI3K, and p‐AKT levels (Figure 7F), accompanied by markedly reduced tumor‐promoting activity (Figure 7G,H; Figure S8N,O).

## 3 Discussion

In this study, we confirmed the tumor‐promoting function of RFC4 in TNBC. RFC4 facilitated tumor growth and metastasis in vivo and enhanced TNBC cell proliferation, migration, and invasion in vitro. Mechanistically, we discovered that TRIM28 acts as a transcription factor for RFC4. Furthermore, TRIM28 interacts with and ubiquitinates RFC4 at K205 and K217 via K29‐linked polyubiquitin chains, thereby enhancing RFC4 protein stability. We also found that RFC4 stabilizes RAP1 and subsequently activates PI3K–AKT signaling. Ultimately, our data confirm that the TRIM28‐RFC4‐RAP1 axis drives TNBC progression via PI3K–AKT signaling (Figure 7H).

Although RFC4 may be involved in tumor progression, its role in cancer remains insufficiently defined, with existing studies largely focused on lung cancer [23]. Here, we identified aberrant RFC4 expression in TNBC and showed that TRIM28 regulates RFC4 through both transcriptional activation and ubiquitination‐mediated stabilization. TRIM28 is well known for its roll in transcriptional control [24] and post‐translational modifications [25]. For example, in gastric cancer, it acts as a transcriptional elongation factor to directly activate *CTTN* transcription, while indirectly elevating CTTN expression by modulating STAT3 and NF‐κB p100, ultimately inducing cytoskeletal rearrangement and metastasis [26]. In bladder cancer, TRIM28 is recruited to mutant promoters to directly activate *hTERT* transcription and neutralizes the transcriptional inhibitory effect of TRIM24 on *hTERT* [27].

Beyond transcription, TRIM28 has attracted considerable attention for its function as a RING‐domain E3 ubiquitin ligase. Wang et al. demonstrated that TRIM28 stabilizes SETDB1 via ubiquitination, thereby maintaining telomere DNA integrity and modulating genomic instability in tumor cells [28]. Additionally, TRIM28 has been reported to promote K63‐linked polyubiquitination of RIPK1, resulting in NF‐κB pathway activation, attenuation of tumor‐induced immune tolerance, and enhanced response to PD‐1 blockade [29]. Intriguingly, our study reveals that TRIM28 mediates the ubiquitination‐dependent stabilization, rather than degradation, of RFC4. While classically associated with degradation, ubiquitination frequently dictates diverse protein fates depending on ubiquitin chain topologies. For instance, the E3 ligase TRAF6 undergoes TGF‐β‐induced autoubiquitination, subsequently promoting K63‐linked polyubiquitination at Lys 34 of TAK1 to activate the TAK1‐p38/JNK cascade and induce cell apoptosis [30]. Furthermore, in non‐small cell lung cancer, TRIM28 mediates the K63‐linked ubiquitination of DDX39A to enhance its expression [31]. Consistent with these non‐degradative functions, we found that TRIM28 catalyzes the assembly of K29‐linked ubiquitin chains on RFC4. Although the biological functions of K29‐linked ubiquitination in oncology remain poorly explored, accumulating evidence confirms its role in protein stabilization. For example, EDD activates the Wnt signaling pathway by promoting K11/K29 polyubiquitination of β‐catenin, thereby enhancing its stability [32]. Similarly, UbcH7 stabilizes p27 and promotes cell migration via K29 polyubiquitination [33]. Mechanistically, K29‐linked ubiquitination may antagonize K48‐linked degradation, as shown by SPOP‐mediated K27/K29‐linked ubiquitination of 17βHSD4, which competes with SKP2‐mediated K48‐linked ubiquitination and thereby stabilizes 17βHSD4 [34]. Therefore, whether TRIM28‐mediated K29‐linked ubiquitination similarly protects RFC4 from degradative ubiquitination warrants further investigation. Together, our findings suggest that TRIM28 promotes RFC4 accumulation through non‐degradative K29‐linked ubiquitination, thereby contributing to TNBC progression.

Notably, our study reveals a dual regulatory mechanism whereby TRIM28 governs RFC4 through both transcriptional activation and post‐translational stabilization. Mutating the enzymatic active site of TRIM28 abolished its ability to stabilize RFC4 via ubiquitination. Although RFC4 expression still increased under these conditions, it remained significantly lower than in the wild‐type group. This finding indicates that TRIM28 maximizes RFC4 expression through the synergistic coupling of transcription and protein stabilization. Analogous dual‐layered regulatory networks have been documented previously. For instance, PARP1 acts as a transcriptional corepressor of *Siah1* while simultaneously facilitating Siah1 protein degradation via ubiquitination, thereby jointly modulating its overall expression [35].

In exploring downstream signaling regulated by RFC4, we focused on its effect on RAP1 protein stability. RAP1, a multifunctional small GTPase of the Ras superfamily, drives tumorigenesis by activating multiple signaling cascades, notably the PI3K–AKT pathway [21, 32, 36]. RAP1 binds directly to the PI3K Ras‐binding domain (RBD), facilitating PI3K activation and subsequent PIP3 accumulation [37]. Our data show that RFC4 enhances RAP1 stability and upregulates its expression, leading to downstream PI3K‐AKT hyperactivation. Although the precise mechanism of RFC4‐mediated RAP1 stabilization remains to be elucidated, previous evidence indicates that RAP1 can be stabilized by USP32 [38], which has been reported to reduce K48‐linked polyubiquitination of NICD1 and thereby increase NICD1 stability [23]. On this basis, we initially hypothesized that RFC4 might stabilize RAP1 by recruiting USP32. However, our validation experiments showed no interaction between USP32 and RFC4. Therefore, identifying the key intermediary factor that mediates the effect of RFC4 on RAP1 stability, as well as the underlying stabilization mechanism, will be a critical focus of future research.

Clinically, although PI3K‐AKT‐mTOR (PAM) pathway inhibitors are approved for HR+/HER2− breast cancers, their application in TNBC remains limited. Despite extensive evidence of aberrant PAM pathway activation in TNBC [39], high tumor heterogeneity and a lack of reliable biomarkers have limited inhibitor efficacy in this subtype. Here, we observed significant RFC4 upregulation in TNBC patient samples and corresponding PI3K‐AKT hyperactivation in RFC4‐overexpressing xenograft models. Crucially, treatment with PI3K‐AKT inhibitors robustly suppressed the pro‐tumorigenic effects induced by RFC4 overexpression. These findings suggest that RFC4 may have potential as a biomarker and that PAM pathway inhibitors may provide therapeutic benefits for TNBC patients with high RFC4 expression. However, given the single‐center TNBC cohort and limited clinical characteristics analyzed in this study, additional multicenter cohorts and TNBC subtype‐stratified analyses are needed to evaluate the independent prognostic value of RFC4.

In conclusion, our findings indicate that TRIM28 drives RFC4 upregulation in TNBC, revealing a novel link between TRIM28, RFC4, and the PAM pathway. Although no targeted inhibitors for TRIM28 are currently available, the essential role of RFC4 in DNA damage repair points to potential therapeutic avenues. Further studies are needed to determine whether the TRIM28–RFC4 axis mediates resistance to chemotherapy, radiotherapy, and PARP inhibitors in TNBC. Together, these findings identify the TRIM28–RFC4–RAP1 signaling axis as a key driver of TNBC progression and a potential target for therapeutic target.

## 4 Materials and Methods

### 4.1 Clinical Samples

Ten pairs of fresh TNBC and matched adjacent normal tissues were obtained from patients undergoing surgical resection without prior radiotherapy or chemotherapy at the Affiliated Hospital of Qingdao University. Additionally, 80 paraffin‐embedded TNBC and matched adjacent normal tissue sections, diagnosed between 2014 and 2016, were collected for retrospective analysis.

### 4.2 Cell Lines and Culture

MCF‐10A, a normal human breast epithelial cell line; the TNBC cell lines MDA‐MB‐231, MDA‐MB‐468, BT‐549, and Hs578t; and HEK‐293T cells were supplied by Haixing Biological Co. (Suzhou, China). MCF‐10A was cultured in Procell specialized medium (Wuhan, China). BT‐549 was cultured in RPMI‐1640 supplemented with 10% fetal bovine serum and 1% penicillin‐streptomycin, whereas the other cell lines were cultured in DMEM with the same additives. All cells were kept at 37°C and 5% CO_2_ atmosphere.

### 4.3 Reagents and Antibodies

The antibodies used for immunoblotting included anti‐TRIM28 (15202‐1‐AP, 1:1000) and anti‐β‐actin (20536‐1‐AP, 1:5000) from Proteintech Group Inc. (Chicago, USA); anti‐RFC4 (A5485, 1:1000) from Abclonal Technology (Wuhan, China); anti‐GAPDH (R1210‐1,1:5000), anti‐HA (0906‐1,1:5000), anti‐FLAG (0912‐1,1:5000), anti‐MYC (R1208‐1,1:5000), anti‐PI3K (ET1608‐70,1:5000), anti‐P‐PI3K (HA721672,1:1000), anti‐AKT (HA721870,1:1000), and anti‐P‐AKT (ET1607‐73,1:1000) were purchased from Hua Bio (Huangzhou, China); and the anti‐RAP1(R389081,1:1000) was obtained from Zen Bio (Chengdu, China). The proteasome inhibitor MG132, protein synthesis inhibitor CHX, autophagy inhibitor CQ and PI3K inhibitor LY294004 were purchased from MedChem Express (Shanghai, China).

### 4.4 Cell Transduction and Transfection

Lentiviruses for RFC4 overexpression and knockdown were obtained from GeneChem (Shanghai, China). Cells were infected for 12 h and selected with puromycin (2 µg/mL) 72 h later to establish stable cell lines. HEK‐293T cells were transfected with plasmids using Lipofectamine 3000 (Invitrogen) according to the manufacturer's instructions. Sequences of siRNAs and lentiviral constructs are provided in Table S1.

### 4.5 Western Blotting (WB)

Cells were lysed in RIPA buffer supplemented with protease and phosphatase inhibitor cocktails. Protein lysates were separated by SDS–PAGE and transferred to PVDF membranes. The membranes were blocked and incubated overnight at 4°C with the indicated primary antibodies, followed by incubation with HRP‐conjugated secondary antibodies for 1 h. Immunoreactive bands were detected using ECL reagent.

### 4.6 RNA Extraction and RT‐qPCR

Total RNA was extracted using TRIzol reagent (Vazyme, China). One microgram of RNA was reverse‐transcribed into cDNA using the PrimeScript RT Kit (Kangwei, China). qPCR was performed using a Life Technologies system, and data were analyzed with QuantStudio software. Relative gene expression was calculated using the 2^−ΔΔCt^ method. Primer sequences are listed in Table S2.

### 4.7 IHC Staining

IHC staining was conducted according to a previously reported protocol [40]. Briefly, tissue sections were incubated overnight at 4°C with an anti‐RFC4 antibody (1:300, ABclonal) and then incubated at 37°C for 30 min with a sheep anti‐rabbit secondary antibody (1:100, Abcam). The staining signal was developed using diaminobenzidine (DAB), and the sections were counterstained with hematoxylin before evaluation.

### 4.8 Immunofluorescence Staining

MDA‐MB‐231 cells cultured in confocal dishes were fixed with 4% paraformaldehyde for 20 min and subsequently blocked with 1% BSA for 1 h. The cells were then incubated with anti‐TRIM28 and anti‐RFC4 antibodies overnight at 4°C, followed by the application of Alexa Fluor 594‐ and Alexa Fluor 488‐conjugated secondary antibodies. Nuclei were stained with DAPI, and fluorescence images were captured utilizing a confocal microscope.

### 4.9 Co‐Immunoprecipitation (Co‐IP)

Cells were lysed in RIPA buffer, and the lysates were centrifuged to collect the supernatants. The supernatants were incubated overnight at 4°C with the indicated primary antibodies and Protein A/G magnetic beads (MedChemExpress). After magnetic separation, the beads were washed three times with PBS, heated in SDS–PAGE loading buffer (NCM Bio) for 10 min, and subjected to western blot analysis.

### 4.10 Chromatin Immunoprecipitation (CHIP)

Cells were treated with 1% formaldehyde for fixation, and the cross‐linking process was halted using glycine. Following sonication, protein–DNA complexes were isolated with an anti‐FLAG antibody. The cross‐links were subsequently reversed, and the resulting enriched DNA fragments were purified and measured using qPCR. Primer sequences are listed in Table S2.

### 4.11 Cell Viability and Colony Formation Assays

For cell viability assays, cells were seeded in 96‐well plates and assessed at 0, 24, 48, and 96 h using CCK‐8 reagent. Ten microliters of CCK‐8 solution was added to each well before measurement. For colony formation assays, cells were seeded in six‐well plates and cultured for 2 weeks. Colonies were then fixed with formaldehyde for 15 min and stained with crystal violet.

### 4.12 Wound Healing Assays

Cells were cultured in six‐well plates until confluent. A linear scratch was made using a 200‐µL pipette tip, and the wells were washed three times with PBS to remove detached cells. The cells were then maintained in serum‐free medium, and images of wound closure were captured at 0 and 24 h.

### 4.13 Migration and Invasion Assays

After 24 h of serum starvation, cells suspended in 100 µL of serum‐free medium were seeded into the upper chambers of Transwell inserts (Corning). Medium supplemented with 10% FBS was added to the lower chambers as a chemoattractant. After 8 h of incubation, cells that had migrated to the lower surface of the membrane were fixed with formaldehyde and stained with crystal violet. Invasion assays were performed similarly, except that the upper chambers were precoated with 80 µL of Matrigel diluted 1:10 in DMEM for 2 h before cell seeding.

### 4.14 Animal Experiments

All animal studies received approval from the Animal Ethics Committee at Qingdao University. A total of ten 4‐week‐old female nude mice (sourced from GemPharmatech, Jiangsu, China) were allowed to acclimatize for a period of 1 week. MDA‐MB‐231 cells (1 × 10^6^) were suspended in 100 µL of PBS and then combined with 100 µL of Matrigel (8.1 mg/mL) before being subcutaneously injected into the right axillary region of each mouse. The sizes of the tumors were measured on a weekly basis using calipers, and the tumor volume was calculated using the following formula: Volume = 1/2 × length × width^2^. Mice were monitored for 6 weeks, ensuring tumor volumes did not exceed ethical limits (1500 mm^3^).

### 4.15 Statistical Analysis

Statistical analyses were performed using GraphPad Prism 8.3.0. Results from a minimum of three separate experiments are shown as the average ± SEM. For comparisons between two groups, two‐tailed Student's *t*‐tests were utilized, while one‐way ANOVA was employed for evaluating differences among multiple groups. The relationship between RFC4 expression and clinicopathological factors was assessed using the Wilcoxon rank‐sum test. Statistical significance was defined as *p* < 0.05 (*p < 0.05, **p < 0.01, ***p < 0.001, ****p < 0.0001).

## Author Contributions

Chengqin Wang and Ceshi Chen conceived the study, and Chen Guo and Li Liu performed the experiments. Lixiang Sun, Litong Liu, Han Wei, Yutong Yan, Fei Yu, Guangming Fu, and Xianning Dong analyzed the data; Cheng Guo, Chengqin Wang, and Ceshi Chen wrote and revised the manuscript. All authors reviewed and approved the final version of the manuscript.

## Funding

This work was supported by Noncommunicable Chronic Diseases‐National Science and Technology Major Project (2023ZD0502200), the National Natural Science Foundation of China (U2102203, 82430084, and 81672606), the Natural Science Foundation of Shandong Province (ZR2022MH206), Yunnan Fundamental Research Projects (202501AS070023), Yunnan Academician Expert Workstation (202505AF350058), and Yunnan International Joint Laboratory of Targeting Breast Cancer Immune Microenvironment (202503AP140011).

## Ethics Statement

The Medical Ethics Committee of the Affiliated Hospital of Qingdao University granted approval for this study (approval no. QYFY WZLL 42060) and the Laboratory Animal Welfare Ethics Committee of Qingdao University (approval no. 20250408BALB/cNu1420250509088). All patients or their legal guardians gave written informed consent.

## Conflicts of Interest

The authors declare no conflicts of interest.

## Supporting information




**Supporting File**: mco271043‐sup‐0001‐SuppMat.docx.

## Data Availability

The information backing the results of this research can be obtained from the corresponding author with a reasonable request.
