# Supplementary material for: TRIM28 Increases RFC4 Protein Expression to Promote Triple‐Negative Breast Cancer Progression
Source: MedComm (2020). 2026 Oct 4;7(10):e71043. doi: 10.1002/mco2.71043 (PMC13635811; doi:10.1002/mco2.71043)
Supplement: Supplementary file 1 [file MCO2-7-e71043-s001.docx]

**TRIM28 increases RFC4 protein expression to promote triple-negative breast cancer progression**

Cheng Guo^1,2#^, Li Liu^3#^, Lixiang Sun^1^, Litong Liu^6^, Han Wei^2^, Yutong Yan^1^, Fei Yu^2^，Guangming Fu^1^, Xianning Dong^1^, Ceshi Chen^4,5*^, Chengqin Wang^1,2*^

^1^ Department of Pathology, the Affiliated Hospital of Qingdao University, Qingdao, Shandong, China

^2^ Department of Pathology, School of Basic Medicine, Qingdao University, Qingdao, Shandong, China.

^3^ Department of Breast and Thyroid Surgery, The First Affiliated Hospital of Chongqing Medical University, Chongqing, China

^4^ Yunnan Key Laboratory of Breast Cancer Precision Medicine, Academy of Biomedical Engineering, Kunming Medical University, Kunming, China

^5^ Yunnan Key Laboratory of Breast Cancer Precision Medicine, The Third Affiliated Hospital, Kunming Medical University, Kunming, China

^6^ The Third People's Hospital of Datong City, Shanxi Province，Shanxi,China

\# These authors contribute equally

* Correspondence to Ceshi Chen, E-mail: [chenc@kmmu.edu.cn](mailto:chenc@kmmu.edu.cn;) or Chengqin Wang, E-mail: [wcq0879@126.com](mailto:wcq0879@126.com)

**Figure S1. RFC4 expression in TNBC.**
(A–B) RFC4 expression levels in TNBC tissues and normal tissues based on the CPTAC database. (C–D) Western blot and RT-qPCR analyses of RFC4 expression in a normal breast cell line, TNBC cell lines, and non-TNBC breast cancer cell lines. *P < 0.05, **P < 0.01, ***P < 0.001.

**Figure S2. RFC4 promotes TNBC cell proliferation, migration, and invasion.**
(A–C) Western blot and RT-qPCR analyses of RFC4 expression in four TNBC cell lines. (D–G) TNBC cells were transduced with lentivirus, and transduction efficiency was validated by western blot analysis. (H–K) Colony formation assays were performed in MDA-MB-468 and Hs578T cells stably expressing shRFC4 or oeRFC4. (L–M) Cell viability was assessed using the CCK-8 assay. (N–P) Wound-healing assays were performed to evaluate cell migration ability. (Q–S) Transwell assays were performed to assess cell migration and invasion abilities; Scale bar, 50μm. *P < 0.05, **P < 0.01, ***P < 0.001.

 **Figure S3. TRIM28 activates the RFC4 promoter region and enhances RFC4 transcription.**
(A–C) Correlation analysis of RFC4 with POU2F2, USF2, and TBP using TIMER2.0. (D) TRIM28 expression in tumors. (E) TRIM28 expression levels across three breast cancer subtypes based on the CPTAC database. (F–H) Expression levels of TRIM28 and RFC4 in TNBC cells. (I–J) Human TFBS prediction identified eight potential TRIM28-binding sites within the RFC4 promoter region. *P < 0.05, **P < 0.01, ***P < 0.001.

 **Figure S4. TRIM28 specifically interacts with RFC4 in TNBC cells.**
(A) Gene interaction network screened using the STRING database. (B) Co-IP analysis confirming the interaction between TRIM28 and RFC4. (C) Predicted binding mode of RFC4 with the TRIM28 homology model generated by AlphaFold. (D–F) Analysis of the half-life of RFC4 protein. **Figure S5. TRIM28 assembles K29-linked ubiquitin chains on RFC4.**
(A) TRIM28 ubiquitinates RFC4 in both nuclear and cytoplasmic compartments. (B) TRIM28 promotes RFC4 ubiquitination through the assembly of K29-linked ubiquitin chains.

 **Figure S6. TRIM28 ubiquitinates RFC4 at K205 and K217.**
(A) The AAA domain of RFC4 is ubiquitinated by TRIM28. (B) Identification of lysine residues within the AAA domain. (C) TRIM28 ubiquitinates RFC4 at K205 and K217.

 **Figure S7. RFC4 activates the RAP1–PI3K–AKT signaling pathway.**
(A) Bubble plot showing the KEGG enrichment analysis of differentially expressed genes. (B) Heatmap showing the enrichment of proteins involved in the RAP1 pathway. (C-D) GSEA analysis showing the association between RFC4 and the RAP1 pathway. (E) Network of the RAP1 pathway. (F–G) Immunoblot analysis of RAP1–PI3K–AKT pathway-related proteins in MDA-MB-231 cells stably expressing shRFC4 or oeRFC4.

**Figure S8. RFC4 accelerates TNBC progression through the RAP1–PI3K–AKT signaling pathway.**
(A) IHC analysis of subcutaneous tumors in nude mice. TRIM28 and RAP1 were predominantly localized in both the nucleus and cytoplasm, whereas p-PI3K and p-AKT were localized in the nucleus, cytoplasm, and cell membrane (400X) ; Scale bar, 50μm. (B–C) Transwell assays were performed to evaluate cell migration and invasion abilities; Scale bar:50μm. (D–E) Colony formation assays were performed to determine the effects of RFC4 and LY294002. (F) mRNA expression levels of RFC4 and RAP1 in TNBC cells. (G–H) Analysis of the half-life of RAP1. (I) Immunoblot analysis of RAP1–PI3K–AKT pathway-related proteins in MDA-MB-231 cells stably overexpressing TRIM28. (J–K) Colony formation assays were performed to determine the effects of TRIM28 and LY294002. (L–M) Transwell assays were performed to evaluate cell migration and invasion abilities; Scale bar, 50μm. (N–O) Effects of RFC4 ubiquitination-site mutations on cell proliferation. *P < 0.05, **P < 0.01, ***P < 0.001.

**Table S1. Sequences of siRNAs and lentiviral constructs**

| **Name** | **Type** | **Sequence（5’-3’）** |
| --- | --- | --- |
| si-TRIM28-1 | siRNA | GCGGAAAUGUGAGCGUGUA |
| si-TRIM28-2 | siRNA | CCAAAGACAUCGUGGAGAATT |
| si-RAP1 | siRNA | TTGGATCTATCAACAGTTACACA |
| shRFC4-1 | shRNA | CCTGACCTCTAGATGTTCAAA |
| shRFC4-2 | shRNA | CAGAAGTCTATTATCACAGAA |
| shRFC4-3 | shRNA | GCATCTGATGAACGTGGAATA |

**Table S2. Sequence of primers used in RT-qPCR and PCR analysis**

| **Name** | **Forward primer (5’-3’)** | **Reverse primer (5’-3’)** |
| --- | --- | --- |
| RFC4 | AGCGACTTCCTGACACAGTTA | TTGGGCCTGAACTTTTCCGAT |
| TRIM28 | GCCTCTACACAGGTCTCACAC | TTTCATGCGTGATAGTGGCAG |
| RAP1 | GGCTTCCACAAGCATCTTTTG | TGAAGGACCGCTACCTCAAG |
| BS1 | AGTTCTGCCTGAAAGTCCGG | ATACGCCGGTAGGAATACGC |
| BS2 | AAGCCACGTGTCCTCAAGAG | GAAGGAGTAGAGGTCGCGC |
| BS3 | ACCAGTAGAGAAGGTGGCCT | GTCTGCGTGTTGTGGAGAGA |
| BS4 | AGCAAATGGATACCATCTATTTTCAT | GTGACAGACAATGTGATGTGTGG |
| BS5 | CTCCTAGTCTGTAAAGCTACTTGAGG | GCAACAAATGCTTACAGAATGTCC |
| BS6 | CGCAAGCCAAAATGGACAAAG | AGGAACAGCACAACCACAGA |
| BS7 | CGCAATTATAACTCCCAAGGTGA | TGCTATGTTTATAAGCCATAGATTGGG |
| BS8 | CGTATACGGGCAAACTCCCT | ATACGCCGGTAGGAATACGC |
